# A generic approach for the development of short-term predictions of *Escherichia coli* and biotoxins in shellfish

**DOI:** 10.3354/aei00265

**Published:** 2018-04-19

**Authors:** Wiebke Schmidt, Hayley L. Evers-King, Carlos J. A. Campos, Darren B. Jones, Peter I. Miller, Keith Davidson, Jamie D. Shutler

**Affiliations:** 1 Centre for Geography, Environment and Society, University of Exeter, Penryn Campus, Penryn TR10 9FE, UK; 2 Plymouth Marine Laboratory, Prospect Place, The Hoe, Plymouth PL1 3DH, UK; 3 Centre for Environment, Fisheries & Aquaculture Science (Cefas), Weymouth Laboratory, Barrack Road, Weymouth DT4 8UB, UK; 4 Scottish Association for Marine Science, Oban, Argyll PA37 1QA, UK

**Keywords:** *Escherichia coli*, Modelling, Forecast, Shellfish, Water quality, Okadaic acid, Dinophysistoxins

## Abstract

Microbiological contamination or elevated marine biotoxin concentrations within shellfish can result in temporary closure of shellfish aquaculture harvesting, leading to financial loss for the aquaculture business and a potential reduction in consumer confidence in shellfish products. We present a method for predicting short-term variations in shellfish concentrations of *Escherichia coli* and biotoxin (okadaic acid and its derivates dinophysistoxins and pectenotoxins). The approach was evaluated for 2 contrasting shellfish harvesting areas. Through a meta-data analysis and using environmental data (*in situ*, satellite observations and meteorological nowcasts and forecasts), key environmental drivers were identified and used to develop models to predict *E. coli* and biotoxin concentrations within shellfish. Models were trained and evaluated using independent datasets, and the best models were identified based on the model exhibiting the lowest root mean square error. The best biotoxin model was able to provide 1 wk forecasts with an accuracy of 86%, a 0% false positive rate and a 0% false discovery rate (n = 78 observations) when used to predict the closure of shellfish beds due to biotoxin. The best *E. coli* models were used to predict the European hygiene classification of the shellfish beds to an accuracy of 99% (n = 107 observations) and 98% (n = 63 observations) for a bay (St Austell Bay) and an estuary (Turnaware Bar), respectively. This generic approach enables high accuracy short-term farm-specific forecasts, based on readily accessible environmental data and observations.

## Introduction

Aquaculture plays a major role in meeting the demand in seafood production, and with the decline of wild fish stocks (FAO 2005), production is expected to grow further (Kobayashi et al. 2015). In Europe, shellfish aquaculture produced 632 000 t of bivalves in 2014, which constituted 25% of the total European marine and coastal aquaculture production (FAO 2016). The sustainability of shellfish farming businesses can be compromised by events of poor water quality due to either microbiological contamination or naturally occurring marine phytoplankton producing biotoxins, both of which can cause temporary closures of shellfish aquaculture harvesting. Frequent or sustained events can often determine the success or failure of an aquaculture business. Furthermore, closure due to poor water quality has been shown to influence the confidence of consumers in shellfish products (Lucas & Southgate 2012). In the European Union, shellfish harvesting areas are required to be classified according to their sanitary quality, on the basis of *Escherichia coli* monitoring in shellfish flesh (EU 2015). The classification determines the likely contamination with viral and bacterial pathogens and determines the extent of post-harvest processing required, before shellfish can be placed on the market for human consumption. Similar monitoring occurs in many other parts of the world (Rees et al. 2010). Increased *E. coli* concentrations in coastal or estuarine water bodies are often related to direct water run-off from urban and agricultural land, or due to sewage overflow entering the water body (Defra 2011). As a result, environmental factors such as rainfall, river flow and solar radiation can amplify or modulate the abundance and distribution of *E. coli* in shellfish waters (Kelsey et al. 2004, Coulliette et al. 2009, Kay et al. 2010).

Some naturally occurring phytoplankton can produce a range of marine biotoxins (Hinder et al. 2011), and once filtered by shellfish, the biotoxins are retained and hence pose a health risk to humans when the shellfish are consumed (Anderson 2014). Therefore, farmed shellfish are also monitored for biotoxins, and once a threshold concentration within the shellfish is exceeded, the harvesting area is closed (EU 2011). One important phytoplankton genus known to produce the biotoxin okadaic acid (OA) and its derivates dinophysistoxins (DTX) and pectenotoxins (PTX) is *Dinophysis* (Reguera et al. 2012). This group of toxins can cause gastrointestinal illness (diarrhetic shellfish poisoning) in humans even when the density of causative organisms is low (Reguera et al. 2012). Production of toxins in *Dinophysis* cells is influenced by intrinsic and genetic factors as well as by the response of the cells to environmental conditions, e.g. physical, chemical and biological factors (Reguera et al. 2012, Anderson 2014, Whyte et al. 2014, Gobler et al. 2017).

Whilst elevated *E. coli* or biotoxin concentrations within shellfish can cause temporary closure of the farm and restrictions on sales, the shellfish themselves are unharmed. Furthermore, leaving the shellfish living in the water allows the contaminants to depurate and dissipate naturally (Egmond et al. 2004, Davidson et al. 2011). Once this has occurred, the shellfish farm and harvesting is re-opened and all stock can be safely sold and consumed. However, once harvested it is often not economically viable, or, depending on the farm type, even feasible, to return the live shellfish to the farmed waters (Morgan et al. 2009, Berdalet et al. 2016). Therefore, any information to guide farm decisions about when to harvest, when not to harvest and when to sell existing harvested stock at wholesale prices can be beneficial to the farm.

Although previous studies have related environmental conditions, such as rainfall, to *E. coli* or biotoxin concentrations (e.g. Kay et al. 2010), the use of models to predict concentrations within shellfish has been limited (e.g. Bougeard et al. 2011). Here, we identified key environmental drivers of *E. coli* and biotoxin concentrations within the shellfish in 2 different shellfish harvesting areas. This information was then used to create short-term (1 wk) forecast models, with the intention that the forecast could be used to inform and support farm management decisions.

## Materials and Methods

### Study areas

#### Coastal bay: St Austell Bay, UK

**Fig. 1:**
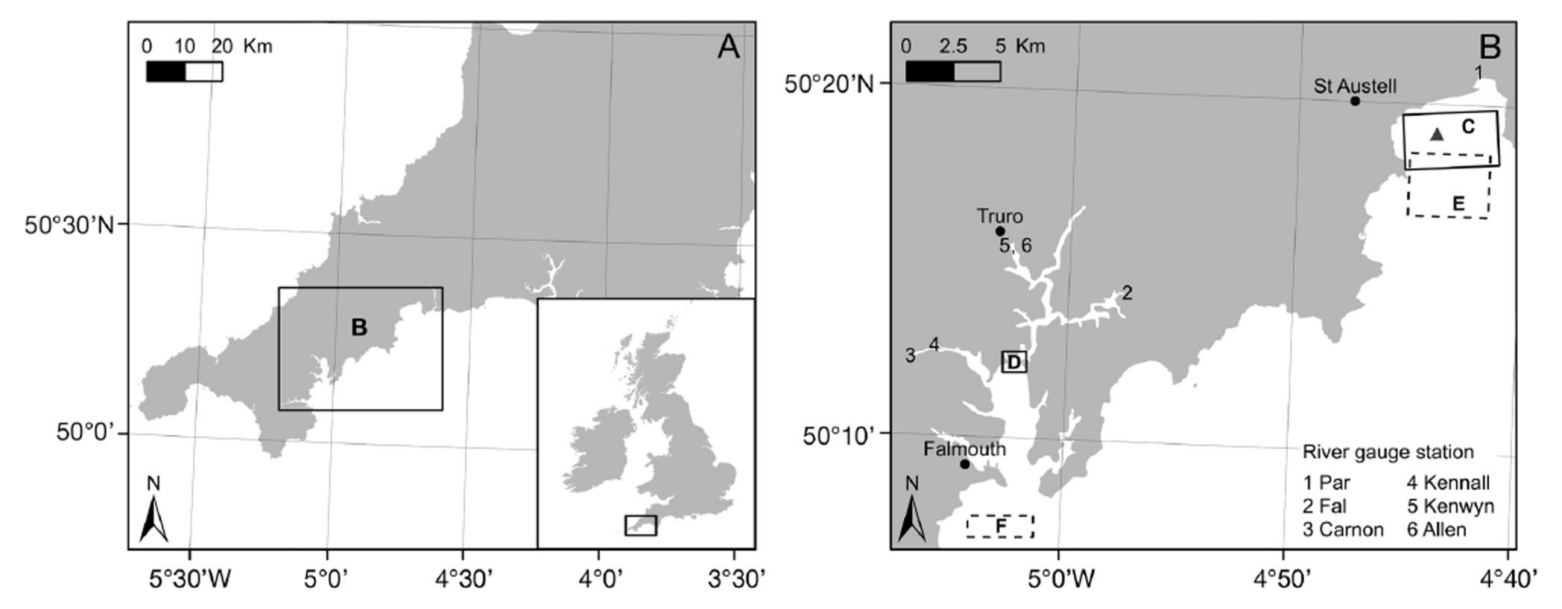
(A) Cornwall, UK, showing (B) details of the study site: location of St Austell Bay (C), Turnaware Bar (D), river gauge stations, *in situ* buoy within St Austell Bay (triangle) and data extraction areas for satellite sea surface temperature (dashed boxes, St Austell Bay: E; Turnaware Bar: F)

St Austell Bay (Fig. 1) is located on the south coast of Cornwall, UK, and covers an area of ~21 km^2^, with mean depth ranges of 5 m near the shore to 20 m at the mouth (Sherwin & Jonas 1994, Sherwin et al. 1997). The bay is characterised by very small tidal currents (about 0.024 m s^−1^), and the circulation within the bay is driven by wind and density effects, with a mean eastwards circulation of ~0.06 m s^−1^ (Sherwin & Jonas 1994). Thermal stratification occurs during calm wind conditions (less than 5 m s^−1^) (Sherwin et al. 1997). The River Par and other smaller streams entering the bay have been noted in the past to influence the near-shore dynamics of the bay (Sherwin et al. 1997). Within the bay, 2 shellfish farms cultivate blue mussels (*Mytilus* spp.) on ropes, and both are classified as class B under the European hygiene classification (EU 2004a).

#### Estuary: Turnaware Bar within the lower Fal estuary, UK

The sheltered Fal estuary is situated at the south coast of Cornwall, UK, and covers an intertidal area of 0.46 km^2^ (ABPmer & HR Wallingford Ltd. 2007). The estuary can be distinguished into 2 geographical areas (Upper and Lower Fal), and this study focussed on Turnaware Bar (Fig. 1), which is within the Lower Fal estuary. For more than 2 centuries, native oysters *Ostrea edulis*, mussels (*Mytilus* spp.) and Pacific oysters *Magallana gigas* (former *Crassostrea gigas*) have been commercially harvested from this estuary. Under European hygiene classification, the bivalve mollusc production area at Turnaware Bar is classified as class B (EU 2004a).

### Data collection and processing

#### Escherichia coli

Following European legislation, concentrations of β-glucuronidase-positive *E. coli* in shellfish (mussels and oysters) are routinely monitored at both study sites. The *E. coli* data were collated for St Austell Bay (sampling site Ropehaven) and Turnaware Bar, for the periods 2008–2016 and 2011–2016, respectively (Table 1). *E. coli* is determined using the enumeration method ISO 16649-3 (ISO 2015), and concentrations are reported as most probable number (MPN) of *E. coli* 100 g^−1^ of flesh. Therefore concentration values at the lower limit of quantification of the MPN method (< 20 MPN 100 g^−1^ and from 2015 onwards <18 MPN 100 g^−1^) were adjusted to 19 and 17 MPN 100 g^−1^, respectively, as recommended by the US National Shellfish Sanitation Program (USFDA & ISSC 2013, 2015). The expanded uncertainty of the *E. coli* MPN method has been estimated at 0.66 (of the log_10_ MPN 100 g^−1^ transformed data), which is calculated as twice the measured standard deviation (SD) (Walker et al. 2018).

#### Biotoxin

Shellfish samples for St Austell Bay are routinely analysed for the group biotoxin OA and its derivates, DTX and PTX, using liquid chromatography coupled with mass spectrometry as described by Cefas (2011). This biotoxin group is reported in µg OA equivalent (eq.) kg^−1^ shellfish flesh, and the Food Standard Agency (FSA) regulatory monitoring data were obtained from Cefas for the time period from 2014–2016 (Table 1). The minimum reporting limit of the analysis method is stated as 16 µg OA eq. kg^−1^ shellfish flesh (EU 2004b,c), and so values within the dataset below this reporting limit were adjusted to 15 µg OA eq. kg^−1^ shellfish flesh.

#### Environmental datasets

A metadata analysis identified that the following variables were important for controlling *E. coli* and biotoxin concentrations within shellfish: rainfall, river flow, solar radiation, sea surface temperature (SST) and wind speed and direction (Šolić & Krstulović 1992, Catalao Dionisio et al. 2000, Izbicki et al. 2009, Raine et al. 2010, Defra 2011, Reguera et al. 2012, Campos et al. 2013). Therefore, a suite of environmental data was collated to allow the linkages between the environmental conditions and the *E. coli* and biotoxin concentrations in shellfish to be investigated. Daily rainfall measurements (mm d^−1^) at Luxulyan (for St Austell Bay) and St Mawes (for Turnaware Bar) were obtained from the UK Environment Agency (EA) for 2008–2016. The rainfall on the day prior to shellfish sampling (lag rainfall) was included as an additional variable. River flow measurements (m s^−1^) of the major rivers (River Par for St Austell Bay; Fal, Carnon, Kennall, Kenwyn and Allen Rivers for Turnaware Bar, Fig. 1) were obtained from the EA for 2008–2016. Due to the multiple river sources, the sum of the river flow of the rivers entering the Lower Fal estuary was calculated and used for the statistical analysis for Turnaware Bar. Reanalysed meteorological forecast data (10 m *U* wind, 10 m *V* wind in m s^−1^ and downwards surface solar radiation in J m^−2^) were downloaded from the ERA-Interim archive for 2008–2015 from the European Centre for Medium-Range Weather Forecast website (http://apps.ecmwf.int/datasets/) (Berrisford et al. 2011). The data for the 2 study sites were then extracted from the nearest model grid point (St Austell Bay: latitude 49.50; longitude 356.25 and Turnaware Bar: latitude 49.50; longitude 354.75). Daily 10 m *U* wind and 10 m *V* wind instantaneous data at 00:00, 06:00, 12:00 and 18:00 h UTC were used to calculate the daily mean wind speed and direction. Daily surface solar radiation at 00:00 and 12:00 h UTC were extracted and added to determine total surface solar radiation d^−1^. Satellite SST observations were obtained for both study sites for 2008–2016. The Multi-scale Ultra-high Resolution (MUR) dataset from the NASA Jet Propulsion Laboratory (http://mur.jpl.nasa.gov/) were used to ensure that data were always available (even in cloudy conditions). These data comprise a daily, 1 km resolution dataset consisting of a combination of infrared and passive microwave-based sensor observations. The mean values between latitude 50.28 and 50.31° N, and longitude 4.69 and 4.75° W were extracted for St Austell Bay, and between latitude 50.12 and 50.13° N, and longitude 5.02 and 5.13° W for Turnaware Bar (Fig. 1). Any pixels defined as land within the MUR land mask were excluded from the analysis.

For the independent evaluation of models and to predict *E. coli* and biotoxin concentrations, near-real time and nowcast environmental data for 2016 for both sites were obtained by the EA and extracted via the MARS ECMWF website (http://apps.ecmwf.int/mars-catalogue/), and satellite data were extracted as described above. The selected dates were chosen to coincide with the official control monitoring results for *E. coli* and biotoxin concentrations tested in shellfish by the FSA and Cefas.

### Environmental drivers for *E. coli* and biotoxin concentrations

#### Statistical approaches for a suite of models

All data exploration and modelling analyses were conducted using the R statistical software (version 3.3.0 on Mac ODS X 10.10.3; R Core Team 2016). The collated datasets were all split into 2, based on 2 time periods. The first dataset was used for model development and initial characterisation of the models. The second dataset was used to provide an independent evaluation of model performance. The details of the dataset splitting are provided in Table 1.

*Generalized linear model development*. Due to the non-Gaussian nature of the response variables (concentrations of OA/DTX/PTX and *E. coli*), they were log transformed prior to use (Kay et al. 2008). Four generalized linear models (GLMs) were developed using the function bestglm() in R. Four different cross-validation methods were used to determine the optimal model (McLeod & Xu 2014). These were (1) R’s default option deleted-*d* cross-validation with random subsamples using the delete-*d* algorithm *d* = ceil{n[1 − 1/(log n − 1)]} and *t* = 10 repetitions, where the parameter *d* is chosen using the formula recommended by Shao (1997); (2) *K*-fold cross validation (Hastie et al. 2009); (3) adjusted *K*-fold cross validation (Davison & Hinkley 1997); and (4) leave-one-out cross-validation approach. To compare the performance of the 4 GLMs to each other, the root mean square error (RMSE) was calculated. The output from R provides a list of the significant explanatory (predictor) variables.

*Averaged GLM development*. The model-averaging GLM based on the information-theoretic approach by Burnham & Anderson (2002) was calculated. Firstly, a ‘global model’ was built, using the glm() function with all environmental factors as dependent variables. In order to directly compare the global model’s independent variables, the model was then standardised (mean = 0 and SD = 0.5). The dredge() function within the R package ‘MuMIn’ (Barto 2015) enabled the generation and comparison of all possible models, and the best performing models, used to construct the model-averaged result, were selected using delta Akaike’s Information Criterion (ΔAIC) <2 as the decision metric. Using the variance inflation factors, the averaged model was tested for potential co-linearity between covariates. Where correlated covariates existed, only 1 variable was retained (Zuur et al. 2010).

*Generalized additive model development*. The gam() function in the R package ‘mgcv’ (Wood 2006) was used to develop the generalized additive model (GAM). Initially, all explanatory variables were set as smooth terms, and knots were set manually to 4, as the number of observations was <100 (Thomas et al. 2015). The estimated degrees of freedom of the smoothed term were used to check the GAM assumptions, and the model terms were then adjusted to be linear terms in cases where estimated degrees of freedom equalled 1 (Thomas et al. 2015).

#### Independent evaluation of all models

An independent comparison is recommended for model evaluation (Verbyla & Litvaitis 1989, Fielding & Bell 1997). Therefore, the accuracy of each model (the best GLM based on its lowest cross-validation RMSE, the best averaged GLM and the GAM) was evaluated using their respective evaluation datasets (Table 1) by calculating the RMSE and bias between the predicted and the actual observed *E. coli* and biotoxin concentrations. The limits and uncertainties in their analytical detection were described above. The best model was identified as the model that produced the lowest RMSE. A confusion matrix analysis was then used to understand how the model accuracy (RMSE and bias) translates to the ability to answer specific management questions. This analysis allows the determination of the accuracy, precision, false positive rate and false discovery rate, which could have significant impacts on an aquaculture business (Stehman 1997). The confusion matrix was used to determine (1) the capability of the best *E. coli* model to predict the EU shellfish bed classification (which is class B for both sites, see above) and (2) the ability of the best biotoxin model to predict the closure and reopening of shellfish harvesting based on the regulatory threshold of 160 µg OA eq. kg^−1^ shellfish flesh.

## Results and Discussion

### Models for *Escherichia coli* concentrations in St Austell Bay and Turnaware Bar

**Fig. 2:**
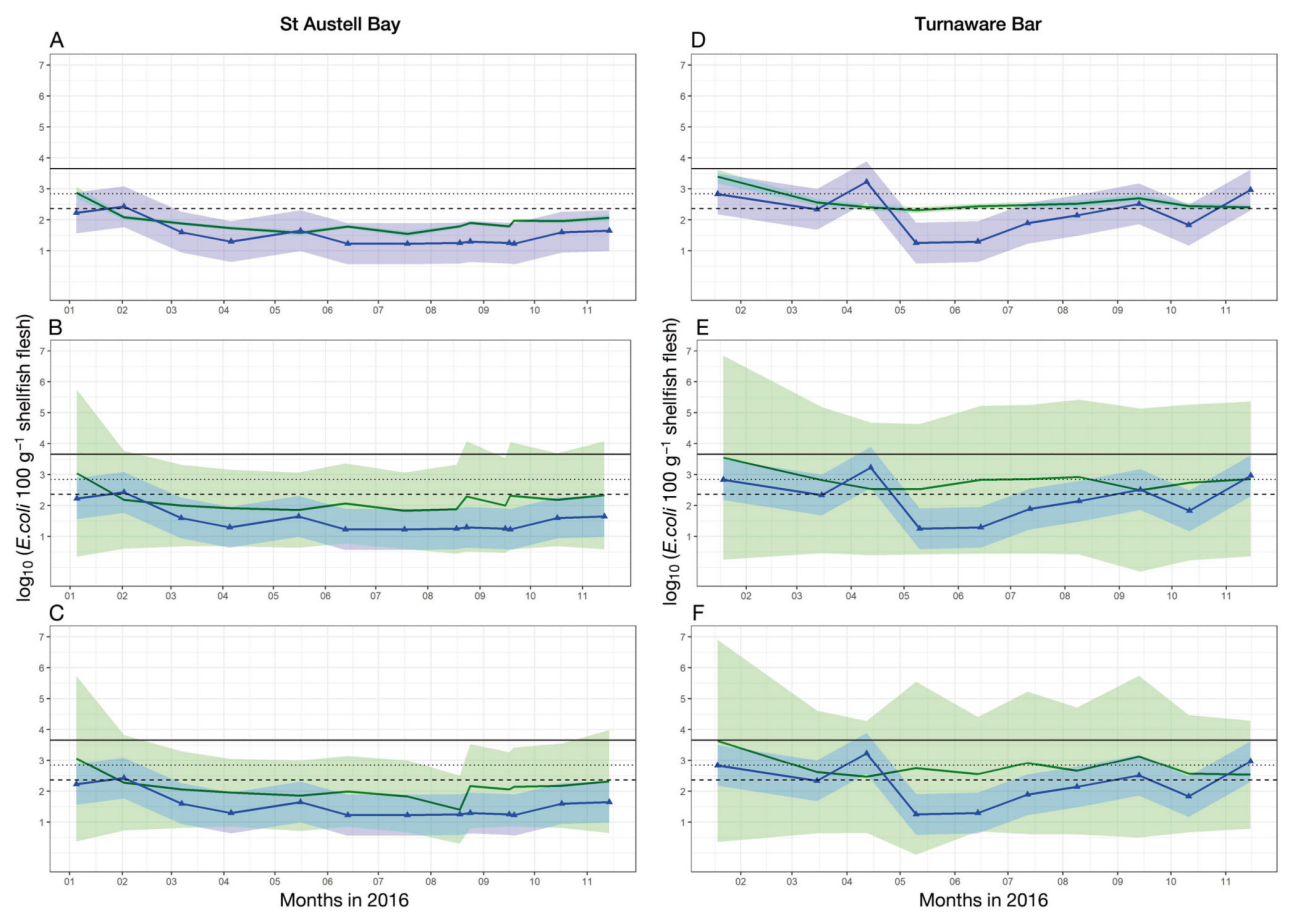
Modelled and observed concentrations of *Escherichia coli* in St Austell Bay and Turnaware Bar for the year 2016. All *E. coli* concentrations are displayed as log_10_ *E. coli* 100 g^−1^ shellfish flesh. Modelled concentrations (green line ± model SE in shaded green) were obtained by (A,D) a generalized linear model (GLM), (B,E) averaged GLM and (C,F) a generalized additive model (GAM). Observed *E. coli* concentrations are shown as a line with triangles (blue line ± uncertainty of 0.66 [2× SD] in shaded blue). The dashed and dotted black lines indicate the limit of class A classification for shellfish bed (dashed = log_10_ 230 *E. coli* 100 g^−1^ shellfish flesh for 80% samples; dotted = log_10_ 700 *E. coli* 100 g^−1^ shellfish flesh for 20% samples), whereas the solid black line represents the upper limit of class B classification (log_10_ 4600 *E. coli* 100 g^−1^ shellfish flesh)

The observed and modelled *E. coli* concentrations are shown in Fig. 2. Both study sites displayed low observed *E. coli* concentrations over the year 2016, with means of 1.54 and 2.23 log_10_ *E. coli* 100 g^−1^ of flesh for St Austell Bay and Turnaware Bar, respectively (Table 2). Observed *E. coli* concentrations were generally below class A limit in St Austell Bay (class A requirements are that 80% of samples must not exceed 230 *E. coli* 100 g^−1^ of flesh and the remaining 20% of samples must not exceed 700 *E. coli* 100 g^−1^ of flesh). Only 1 result during the winter of 2016 exceeded the class A limit of *E. coli* 100 g^−1^ of flesh (Fig. 2; on 2 February 2016). A slightly larger variability in observed *E. coli* concentrations was apparent at Turnaware Bar (Fig. 2).

All 3 model outputs (GLM, average GLM and GAM) fitted the observed *E. coli* concentrations reasonably well. Statistical measures for observed (mean, SD) and modelled (RMSE, bias) *E. coli* concentrations in St Austell Bay and Turnaware Bar are listed in Table 2. For both study areas, the GLM showed the lowest RMSE (St Austell Bay: 0.48 log_10_ *E. coli* 100 g^−1^ shellfish flesh; Turnaware Bar: 0.68 log_10_ *E. coli* 100 g^−1^ shellfish flesh; Table 2).

### Environmental drivers for *E. coli* concentrations in St Austell Bay and Turnaware Bar

Significant explanatory variables used for predicting *E. coli* concentrations for each model and for both study sites are summarised in Table 3. The majority of models identified rainfall and/or lag rainfall as one of the key explanatory variables for predicting *E. coli* concentrations (Table 3).

Rainfall has been previously described as a common environmental factor associated with controlling *E. coli* concentrations in receiving waters (Kelsey et al. 2004, Coulliette et al. 2009, Kay et al. 2010, Campos et al. 2013). However, the degree of response of *E. coli* concentrations to rainfall can vary considerably between sampling points in a given sampling area (Defra 2011), which can be attributed to differences in land use (e.g. agriculture run-off versus urban sewage) and soil conditions surrounding the sampling site (Kay et al. 2010, Defra 2011). In all models for Turnaware Bar, river flow was identified as significant, and this catchment has been previously described as highly responsive to rainfall (Cefas 2012). River flow may also provide a proxy for other events contributing to faecal contamination (e.g. combined sewer overflows, application of slurry to fields, etc.) and therefore it is not possible to explain the direct cause for the varying *E. coli* concentrations. Turnaware Bar, the study point within the Lower Fal estuary, is exposed to a lower ‘flushing’ time due to tides, thus resulting in a potentially higher retention time of microbial contamination from nearby sources (Uncles et al. 2002, Langston et al. 2006).

For St Austell Bay, solar radiation was identified as an important explanatory variable for predicting *E. coli* concentrations (Table 3). All models displayed the solar radiation term as negative, and this is consistent with previous work by Campos et al. (2013) that showed that solar radiation can influence the die-off of *E. coli* in seawater. Additionally, Šolić & Krstulović (1992) suggested that solar radiation may be more important than seawater temperature in altering the number of the faecal indicator. However, for Turnaware Bar, all 3 models identified SST as a key explanatory variable. Although *E. coli* optimal growth temperature lies at around 37°C, it has been previously shown that the optimal temperature for survival is not necessarily the same as that needed for optimal growth (Rozen & Belkin 2001), as *E. coli* can survive and stabilise at lower temperatures within estuaries (Rhodes & Kator 1988).

### Ability to predict shellfish bed classification

Using easily attainable environmental measurements to predict the shellfish *E. coli* concentrations would enable local authorities to estimate the likely variation in *E. coli* within a shellfish area, in the absence of regular monitoring, e.g. if shellfish beds are currently not used, but are available for lease. Therefore, the confusion matrix allowed the overall performance (termed accuracy) and precision (if predicted class B, how often was the model correct) to be calculated for both sites, using the best *E. coli* model (the model with the lowest RMSE).

The overall accuracy for St Austell Bay was 99%, determined using data from 2008–2016 (n = 107), and 100% for data from 2016 only (n = 13). Additionally, the precision was 100% for data from 2008–2016 (n = 107) and 100% for data from 2016 (n = 13). Similarly, results for Turnaware Bar showed a high accuracy of 98% for data from 2011–2016 (n = 63) and 100% using data from 2016 only (n = 10), with a precision of 100% (2011–2016, n = 63) and 100% for just 2016 (n = 10). We note that despite the use of long-term datasets (St Austell Bay: 9 yr; Turnaware Bar: 5 yr), relatively limited variations in *E. coli* are apparent.

### Models for biotoxin concentrations in St Austell Bay

**Fig. 3:**
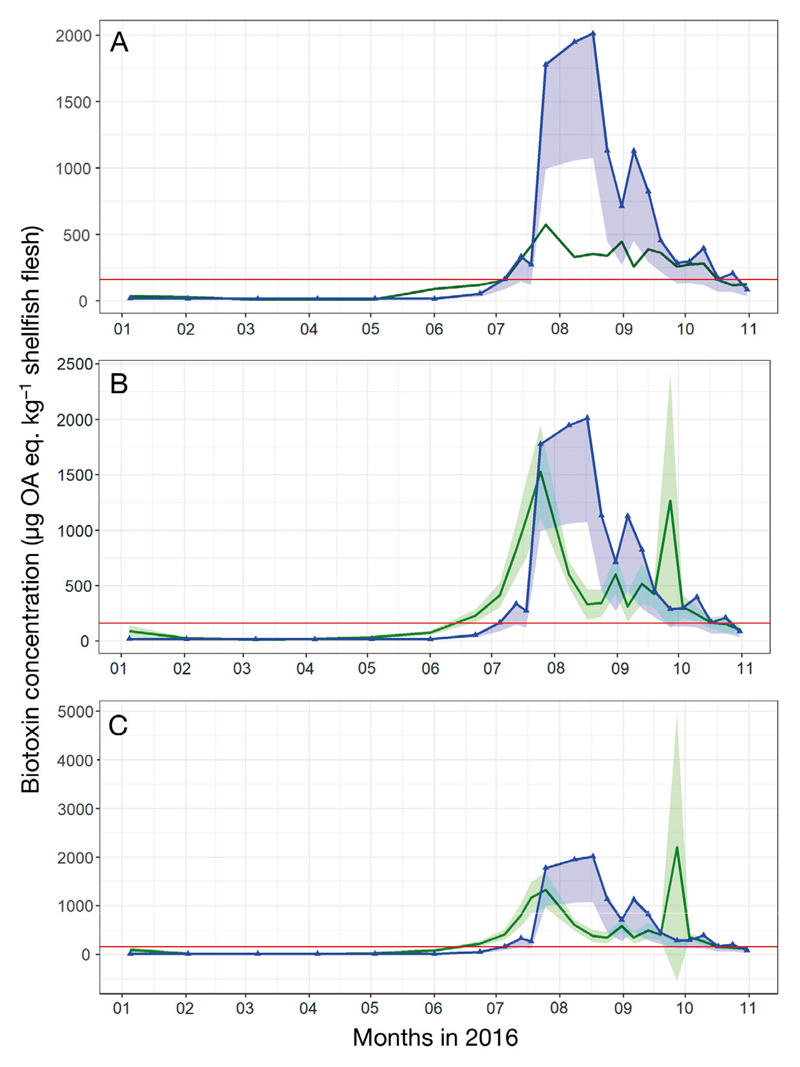
Modelled and observed biotoxin concentrations in St Austell Bay for the year 2016. All biotoxin concentrations are displayed as µg okadaic acid (OA) eq. kg^−1^ shellfish flesh. Modelled concentrations (green line ± model SE, shading) were obtained by (A) a generalized linear model (GLM), (B) averaged GLM and (C) a generalized additive model (GAM). Observed biotoxin concentrations are shown in lines with triangles, where the blue line (shading) = high (low) measurement uncertainty values. The red line indicates the maximum permitted level of 160 µg OA eq. kg^−1^ shellfish flesh at which point the farm is closed. Note different *y*-axis scales; model SE of GLM (in A) were too small for shading

The observed and modelled biotoxin concentrations can be seen in Fig. 3. During 2016, the observed biotoxin concentrations ranged from below the reporting limit up to 2013 µg OA eq. kg^−1^ shellfish flesh. Between mid-July and the end of October, the observed biotoxin concentrations were above the maximum permitted level of 160 µg OA eq. kg^−1^ shellfish flesh (EU 2011), indicated as the red horizontal line in Fig. 3, and therefore the shellfish farm was closed for this period.

Table 4 lists statistical measures for observed (mean, SD) and modelled (RMSE, bias) biotoxin concentrations. The averaged GLM produced the lowest RMSE (582.3 µg OA eq. kg^−1^ shellfish flesh), whereas the GAM showed the smallest bias of –71.01 µg OA eq. kg^−1^ shellfish flesh.

All 3 models were consistent in capturing the increase and reduction of biotoxin concentrations within the shellfish (i.e. the points at which concentrations increase and decrease in relation to the legal limit, Fig. 3). However, all models showed differences in their response when modelling the period between the ‘accumulation’ and ‘depuration’ phases. The depuration of biotoxin concentrations within the shellfish depends upon the species and the physiological conditions of the shellfish, e.g. fat content, respiration state, growth, food availability etc. (Hallegraeff 1995). No physiological parameters, describing the depuration of the group biotoxin OA/DTX/PTX in blue mussels, were found in the published literature, and thus it was not possible to identify specific depuration parameters for the model development. Future studies describing the toxicological profile of OA/DTX/PTX in blue mussels (*Mytilus* spp.) would therefore be beneficial.

### Modelling biotoxin accumulation and depuration phases in St Austell Bay

To investigate the difference between accumulation and depuration of the biotoxin, the models were retrained using either only accumulation data (n = 27 observations from 2014 and 2015) or only depuration data (n = 27 observations from 2014 and 2015). The ‘accumulation’ phase was defined as the beginning of the year to the highest observed biotoxin concentrations, while the ‘depuration’ phase was defined as from the highest observed concentration to the end of the year.

The accumulation models showed higher RMSE (in µg OA eq. kg^−1^ shellfish flesh: GLM = 1745.73; averaged GLM = 858.33; GAM = 1151.99) than the models trained on the full dataset (see above) when evaluated using the full evaluation dataset (Table 4). Similarly, the depuration models also showed a higher RMSE (in µg OA eq. kg^−1^ shellfish flesh: GLM = 868.39; averaged GLM = 110.29; GAM = 17965.03), and both models were unable to capture the appropriate response of the biotoxin concentration. This result supports the usage of the model trained on the full dataset in order to forecast biotoxin concentrations and highlights that different environmental and physiological factors are controlling the accumulation and depuration phase.

### Ability to predict farm closure and reopening due to biotoxins

The ability for a farm to identify the potential closure due to accumulation of biotoxins in mussels is advantageous for supporting decisions on harvesting and sales (e.g. when to sell existing stock at wholesale prices). Therefore, the performance of the model with the lowest RMSE (averaged GLM) trained on the full dataset (Tables 1 & 4) to accurately forecast the closure of a shellfish farm was evaluated. The overall performance (accuracy), false positive rate (false prediction of an open farm, when it should actually be closed) and false discovery rate (error in predicting the opening and closure of the farm) were calculated.

The nowcast predictions (n = 78 observations for 2014–2016) of the confusion matrix produced an accuracy of 84% with a false positive rate of 2% and false discovery rate of 4%. Results using data from just 2016 showed a higher accuracy (92%) with a false positive rate of 6% and a false discovery rate of 13% (n = 24 observations). For a 1 wk forecast (n = 78 observations for 2014–2016), the accuracy was 86% with a false positive rate of 0% and false discovery rate of 0%; in comparison, results for the 1 wk forecast using just 2016 data showed an accuracy of 96%, with a false positive rate of 0% and false discovery rate of 0% (n = 24 observations).

#### Environmental drivers for biotoxin concentrations in St Austell Bay

Environmental variables such as SST, solar radiation and wind speed were identified as key drivers for the different biotoxin models (Table 5). Additionally, other environmental variables, including lag rainfall and wind direction contributed to some of the models.

**Fig. 4:**
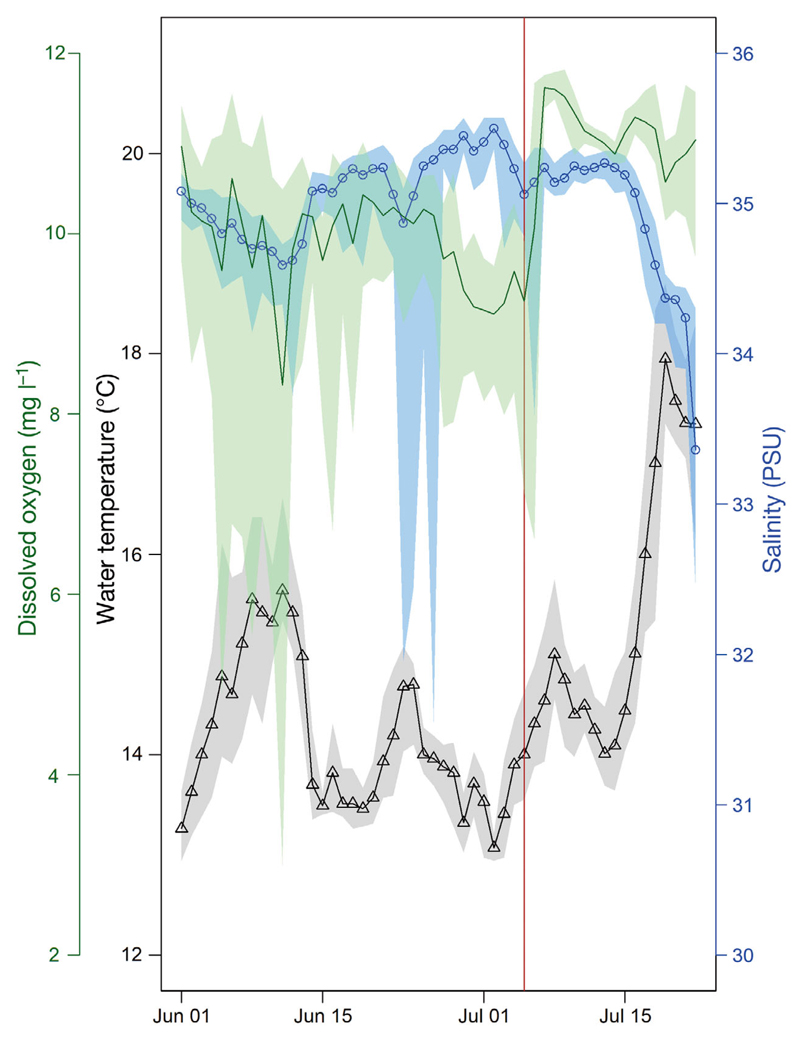
*In situ* data from the monitoring buoy (see Fig. 1) from 1 June to 22 July 2016, with a sea surface temperature (SST) sensor at 1.10 m, a salinity sensor at 1.25 m and an oxygen sensor at 1.65 m depth. The monitoring buoy was located close to the shellfish farm and its design is described in Schmidt et al. (2018). Vertical red line indicates the date (5 July 2016) when the shellfish farm was closed due to high biotoxin concentrations (>160 µg okadaic acid [OA] eq. kg^−1^ shellfish flesh). The mean SST is shown as a black line with triangles, mean salinity is shown as a blue line with circles, and the mean dissolved oxygen concentration is shown as a green line; for all lines, shading represents the minimum and maximum values

Ambient temperature influences the filtration rate and pumping activity in blue mussels (Jørgensen et al. 1990). The distribution and occurrence of the dinoflagellate genus *Dinophysis* spp. in the water column in temperate regions can be related to stratification of the water column (Raine & McMahon 1998). Seawater temperature, salinity and dissolved oxygen concentrations were recorded in close proximity of the shellfish farm from autumn 2015 until summer 2016 using a moored buoy (refer to Schmidt et al. 2018 for its design; triangle in our Fig. 1B). Fig. 4 shows that the SST just outside of the farm at 1.1 m depth increased by 2°C from 2–8 July 2016, indicating that thermal stratification could have taken place within the farm in July (e.g. as the farm itself is likely to accelerate stratification by dampening vertical mixing). In addition, the increased dissolved oxygen concentrations (9.2 to 11.6 mg l^−1^; green line in Fig. 4) from 5–7 July 2016 indicate an increase in biological activity close to the farm site. This period would coincide with the closure of the farm on 5 July 2016 due to high biotoxin concentrations (indicated as a red vertical line in Fig. 4). These observations support the hypothesis (Farrell et al. 2012) that increased abundance of *Dinophysis* spp. in the water column can be related to thermal stratification. However, a future study would need to confirm this by placing the instruments within the mussel farm ropes.

A further important factor influencing the distribution of *Dinophysis* spp. can be transport by ocean currents from offshore locations into coastal bays (Escalera et al. 2010). Raine et al. (2010) suggested that wind-driven water exchange between the south coast of Ireland and the continental shelf is responsible for an influx of *Dinophysis* spp. into a shellfish harvesting area. Large-scale, wind-driven advective processes were also proposed by Whyte et al. (2014) to explain large coastal blooms of *Dinophysis* spp. in the Shetland Islands. All 3 biotoxin models identified wind speed as a key environmental factor for the shellfish biotoxin concentration. However, no significant correlation was found between increased biotoxin concentrations and wind speed or direction. As a third key environmental driver, solar radiation was identified as an important factor by the models. Toxicological profiles of diarrhetic shellfish poisioning (DSP) toxin reported that its synthesis requires light and is coupled to the cell division cycle (Pan et al. 1999). This again supports the hypothesis that stratification is important, as stratification will imply reduced turbidity and therefore an improved light field within the water column. We chose to focus on OA/DTX/PTX toxins, as these were a significant issue for the shellfish farmer in St Austell Bay. Mussels were the focal shellfish, as they dominate the global bivalve production and can be used as indicator species for monitoring the production of other bivalves. Clearly the generic nature of the approach means that it could be applied to other toxin groups (e.g. paralytic shellfish poisoning [PSP], amnesic shellfish poisoning [ASP], azaspiracids poisoing [AZA]) if sufficient data to train and evaluate the models exist. However, for our study site and temporal period (2014–2016), neither PSP nor ASP exceeded the maximum permitted limit, and there were only 3 instances of AZA exceeding the permitted limit. Therefore, we were unable to test the applicability to other toxin groups.

## Conclusions

This study has demonstrated that a generic approach and a suite of readily available environmental data (*in situ*, satellite observations and meteorological nowcasts and forecasts) can be used to successfully model the *Escherichia coli* concentrations within shellfish living in an estuarine and a coastal shellfish site, despite the sites exhibiting contrasting hydrography. The same methodology was then shown to successfully model the biotoxin concentrations within shellfish living in a coastal shellfish site. The accuracy of the models was evaluated and characterised using data independent from the training data, and indirectly verified using measurements from an *in situ* monitoring buoy located close to one of the sites. The inputs to these models were identified by a metadata analysis as being important for influencing the *E. coli* and biotoxin concentrations within shellfish, and the parameters identified as significant by each model analysis are consistent with previous studies.

Whilst the models were less able to accurately predict the absolute values of the concentrations within the shellfish, the modelled variations in *E. coli* and biotoxin concentrations have been demonstrated to be reliable for supporting farm decision making. Accurate classifications of a change in shellfish bed class and forecasting closure due to biotoxin accumulation were both possible. The biotoxin models can be used to provide a 1 wk forecast. Such an early warning can provide and support the shellfish farmers in their management by guiding harvesting decisions and pricing strategies. Using these forecasting approaches is also likely to help increase customer confidence in shellfish products, and give farmers increased confidence when selling their product. After initial model development for a harvesting site, the shellfish farmer could use this approach and the subsequent models to forecast changes within their farm shellfish stock, towards supporting farm management decisions. However, it is likely that a yearly update of the model parameters would be needed to account for temporal changes in the catchment, regional climate, changes to farm composition and size, and influences that the farm itself will have upon the local ecosystem.

## Figures and Tables

**Table 1:** Overview of datasets used for the development and evaluation of the most suitable model for predicting *Escherichia coli* and biotoxin concentrations in farmed shellfish at 2 contrasting coastal sites (see Fig. 1). No. obs.: number of observations

Site	Type	Development		Evaluation	
		Time period	No. obs.	Time period	No. obs.
St Austell Bay	*E. coli*	2008–2015	94	2016	13
	Biotoxin	2011–2015	54	2016	24
Turnaware Bar	*E. coli*	2014–2015	53	2016	10

**Table 2:** Comparison between modelled and observed *Escherichia coli* for 2016 for St Austell Bay and Turnaware Bay according to statistical measures. All values are presented as log_10_ *E. coli* 100 g^–1^ shellfish flesh. RMSE: root mean square error, GLM: generalized linear model; GAM: generalized additive model; *: best performing model

	St Austell Bay				Turnaware Bar			
	Mean	SD	RMSE	Bias	Mean	SD	RMSE	Bias
Observations	1.54	0.39			2.23	0.68		
GLM*	1.92	0.33	0.48	–1.54	2.57	0.31	0.68	0.34
Averaged GLM	2.15	0.32	0.7	0.61	2.82	0.3	0.87	0.58
GAM	2.1	0.37	0.65	0.57	2.79	0.36	0.87	0.56

**Table 3:** Explanatory variables identified for *Escherichia coli* models for St Austell Bay and Turnaware Bar. An asterisk (*) marks variables that contributed significantly (p < 0.05) to the model. GLM: generalized linear model; GAM: generalized additive model; SST: sea surface temperature

Model	St Austell Bay	Turnaware Bar
GLM	Lag rainfall* and solar radiation	River flow* and SST*
Averaged GLM	Lag rainfall*, solar radiation*, rainfall, wind speed and SST	River flow*, SST*, wind direction, solar radiation and rainfall
GAM	Lag rainfall, solar radiation and as smoothed term: rainfall	River flow*, SST* and as smoothed term: lag rainfall*

**Table 4:** Comparison between modelled and observed biotoxin concentrations for 2016 for St Austell Bay according to statistical measures. All values are presented as µg okadaic acid eq. kg^–1^ shellfish flesh. RMSE: root mean square error, GLM: generalized linear model; GAM: generalized additive model; *: best performing model

Observation/model	St Austell Bay			
	Mean	SD	RMSE	Bias
Observed data	514.71	633.46		
GLM	227.61	159.48	596.14	−287.1
Averaged GLM*	404.07	409.7	582.3	−110.64
GAM	443.7	507.24	671.59	−71.01

**Table 5:** Explanatory variables identified for modelled biotoxin concentrations in St Austell Bay. An asterisk (*) marks variables that contributed significantly (p < 0.05) to the model. GLM: generalized linear model; GAM: generalized additive model, SST: sea surface temperature

Model	Explanatory variables for biotoxin in St Austell Bay
GLM	SST* and wind speed
Averaged GLM	SST*, solar radiation*, wind speed, lag rainfall and wind direction
GAM	SST*, solar radiation*, wind speed* and as smoothed term: lag rainfall
